# Deep learning models for predicting opaque bubble layer morphology of keratorefractive lenticule extraction before laser scanning

**DOI:** 10.1016/j.aopr.2026.02.003

**Published:** 2026-02-13

**Authors:** Yifeng Yu, Xuan Wu, Fu Gui, Xinghui Huang, Yunwei Hu, Mengyun Zhou, Xu Huang, Ling Ling, Zeyu Zhu, Tao Zhan, Chong Ai, Yalin Lu, Jingjing Xu, Kang Yu, Fen Xiong, Xinyu Xia, Ding Shangguan, Yugen Yi, Jian Xiong

**Affiliations:** a Ophthalmic Center, The Second Affiliated Hospital, Jiangxi Medical College, Nanchang University, Nanchang, China; b School of Software, Jiangxi Normal University, Nanchang, China; c Guangdong Second Traditional Chinese Medicine Hospital, Guangzhou, China; d Jiangxi Medical College, Nanchang University, Nanchang, China; e Hangzhou Huaxia Eye Hospital, Hangzhou, China; f Nanchang Bright Eye Hospital, Nanchang, China; g The Affiliated Eye Hospital, Jiangxi Medical College, Nanchang University, Nanchang, China; h The Affiliated Hospital of Jiangxi University of TCM, Nanchang, China; i Nanchang Key Laboratory for Blindness and Visual Impairment Prevention Technology and Equipment Development, Nanchang, China

**Keywords:** Keratorefractive lenticule extraction, Deep learning, Intraoperative complication, Opaque bubble layer

## Abstract

**Purpose*s*:**

To develop deep learning (DL) models for predicting opaque bubble layer (OBL) morphology and area before femtosecond laser scanning in keratorefractive lenticule extraction (KLEx) procedures.

**Methods:**

A total of 10276 frames from 5138 KLEx surgical videos, involving 2698 patients, were used to construct and validate the DL models. Suction-initiated frames captured before laser scanning were used as input to construct an OBL perceptual attention network (OBLPA-Net) and a DL-based OBL prediction regression model, which were developed to predict the OBL morphology and area in posterior lenticular cut frames during laser scanning. The performance of models was primarily evaluated by Dice coefficient, intersection over union (IoU), and mean absolute error (MAE).

**Results:**

The OBLPA-Net demonstrated strong predictive performance for OBL morphology, achieving a Dice coefficient of 0.918 (95% confidence interval (CI): 0.915−0.921) and an IoU of 0.849 (95% CI: 0.844−0.854) on the validation set. Good generalizability was observed across external test sets. Moreover, to predict the global quantitative OBL measurement (OBL area in cornea), the regression model yielded an MAE of 0.221% (95% CI: 0.201−0.242) in the validation set, with similar results in external test sets.

**Conclusions:**

Developing accurate prediction models for intraoperative complications in KLEx across surgical steps is feasible and may aid in refractive surgery decision-making, improve surgical techniques, and enhance surgical education.

## 1 Introduction

Myopia is the most common refractive error globally and a significant public health concern due to its rising prevalence.^1^^,^^2^ Laser refractive surgeries, such as Keratorefractive lenticule extraction (KLEx), a newer femtosecond laser technique, are effective treatments offering excellent visual outcomes and safety.^3^ While KLEx has advantages over laser in situ keratomileuses (LASIK), it is technically more challenging and requires a steeper learning curve.^3^, ^4^, ^5^, ^6^ The lack of a standardized re-KLEx procedure adds complexity, particularly in the lenticule creation stage,^5^^,^^7^ making it essential to identify potential complications before laser scanning begins.

One common complication during femtosecond laser ablation is opaque bubble layer (OBL), caused by bubble accumulation in the intrastromal interface, leading to opacification.^5^ This can delay visual recovery and complicate surgical procedures by obscuring the visual axis.^5^^,^^8^ Although risk factors for clinically significant or severe OBL have been identified,^8^, ^9^, ^10^ no model currently predicts its size, location, or morphology.

Decision-making in surgery is both crucial and challenging, particularly when managing procedures and addressing complications. These decisions are vulnerable to cognitive biases, especially when surgeons are inexperienced or fatigued.^11^ Deep learning (DL), a branch of artificial intelligence (AI), effectively processes high-dimensional data and images by adjusting weights in neural networks.^12^ DL plays a vital role in automating medical analyses and managing complex data sets related to diseases.^11^^,^^13^ In surgery, DL is transforming preoperative and intraoperative decision-making, optimizing processes from laparoscopic video analysis to managing extensive patient databases.^13^

In KLEx surgery, the lenticule creation phase, which is entirely machine-controlled, can present complications that often require halting the procedure and opting for alternative techniques.^7^ These complications not only challenge surgeons but also impact the psychological well-being and recovery outcomes of patients.^9^^,^^14^ To mitigate these risks, it is essential to anticipate issues during the suction-initiated step, which allows surgeons to stop and restart the procedure without affecting the laser scanning. Studies suggest that preoperative corneal parameters are key risk factors for OBL during KLEx surgery.^8^, ^9^, ^10^ This raises the question of whether DL can use suction-initiated frames, which contain real-time corneal information, to predict OBL across surgical stages before laser scanning. To address this, we developed a DL system that predicts the morphology and area of the OBL in KLEx surgery using video frames. The system was validated in external clinics, and focused on the posterior lenticular cut frame, where OBL is more challenging to dissect.^8^^,^^14^ These predictions may help surgeons decide if re-suction is necessary, thus improving surgical decision-making.

## 2 Methods

### 2.1 Study design

A retrospective observational study was conducted using data from the Second Affiliated Hospital of Nanchang University, Hangzhou Huaxia Eye Hospital, and Nanchang Bright Eye Hospital. This study was approved by the ethics committee of the Second Affiliated Hospital of Nanchang University (2023 No. (96)); the two collaborating centers, lacking their own institutional review boards, formally agreed to adhere to this central ethical oversight. All participants provided informed consent before enrollment, in accordance with the Declaration of Helsinki. The study followed the Transparent Reporting of a Multivariable Prediction Model for Individual Prognosis or Diagnosis (TRIPOD) reporting guidelines, specifically for the prediction of intraoperative complications.

### 2.2 Surgical procedure

KLEx surgeries were performed by five surgeons across three hospitals (FG, Jian X, Yifeng Y at the Second Affiliated Hospital of Nanchang University; WZ at Nanchang Bright Eye Hospital; Xu H at Hangzhou Huaxia Eye Hospital) using the 500-kHz VisuMax femtosecond laser system (Carl Zeiss Meditec, Jena, Germany) with standardized settings (1043 nm wavelength, 130 nJ energy, 4.5 μm spot and track distance, cap diameter of 7.6 mm). After suction and laser scanning, the posterior and anterior lenticular surfaces were dissected, followed by lenticule extraction through a corneal incision. The postoperative care included 0.5% levofloxacin (Santen Pharmaceutical, Ikoma, Nara, Japan) and 0.1% fluorometholone (Fluorometholone; Santen, Osaka, Japan) drops four times a day for one week.

### 2.3 Database construction

This multi-center study collected KLEx surgery videos from June 15, 2021, to October 11, 2023, along with preoperative ophthalmologic indices, including manifest and cycloplegic refraction and intraocular pressure (CT-80A; Topcon, Tokyo, Japan). Corneal parameters, such as horizontal diameter, central keratometry, and central corneal thickness (CCT), were measured using a corneal topographer (Pentacam HR, OCULUS, Wetzlar, Germany). Optical zone, residual stromal thickness (RST), and lenticule thickness were calculated using VisuMax software.

The inclusion criteria comprised: (1) age 18−45 years; (2) spherical refraction < -10.00 diopters (D) and myopic astigmatism <3.00 D; (3) corrected distance visual acuity of ≥20/25 before KLEx surgery; (4) refractive errors stable for at least 1 year; and (5) CCT ≥480 μm and RST ≥280 μm. The exclusion criteria were: (1) history of ocular trauma or surgery; (2) corneal abnormalities such as keratoconus, severe dry eye, and active ocular diseases; and (3) systemic diseases such as central neurological diseases, hyperthyroidism, and autoimmune diseases.

### 2.4 Data preparation and quality control

**Supplementary Fig. S1:**
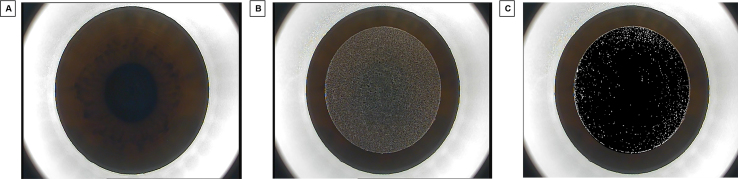
Keyframes of the keratorefractive lenticule extraction videos A, suction-initiated frame. B, posterior lenticular cut frame. C, superbright opaque bubble layer (OBL) image.

Three ophthalmologists (YH, TZ, and FX), each with over three years of clinical experience, independently extracted frame pairs in BMP format from KLEx surgery videos. The suction-initiated frame was defined as the moment when the cornea was fully suctioned and stabilized by the contact glass, immediately prior to laser emission. The posterior lenticular cut frame was captured upon the completion of the posterior plane scanning and lenticule side cut, marked by the appearance of the high-density OBL rim (Supplementary Fig. S1). Any discrepancies in frame selection were arbitrated by a senior surgeon (Jian X). Poor-quality frames (1.8%) were excluded.

**Supplementary Fig. S2:**
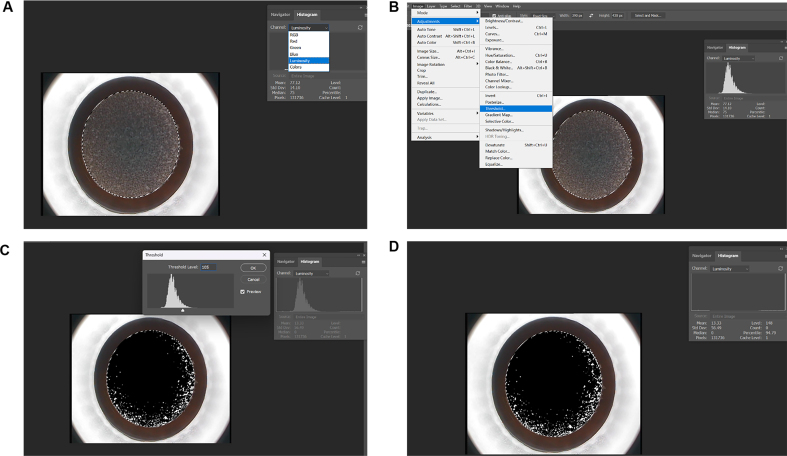
Measurement of the OBL area in cornea A, Using Photoshop software (Adobe Systems, San Jose, CA), the optical zone of cornea was selected using the elliptical marquee tool (380 ∗415 pixels), then the luminosity channel from the histogram window was chosen to record the mean and standard deviation values (Red box). B, Selecting the threshold value. C, Inputting the luminosity threshold calculated by adding two standard deviation values to the mean luminosity value. D, Calculating the OBL-Area [100% - Percentile (Red box) %]. Abbreviations: OBL = opaque bubble layer.

Subsequently, Adobe Photoshop (version 21.2.1, Adobe Systems, San Jose, CA) was used to measure the OBL area within the corneal area selected by the elliptical marquee tool (Supplementary Figure S2). OBL was defined as the percentage of pixels two standard deviations (SD) brighter than the average background. This threshold was selected to standardize the annotation of OBL boundaries, based on the statistical empirical rule for identifying high-intensity signals (distinguishing hyper-reflective anomalies from the 95% background distribution) and its consistency with established literature.^8^ Finally, each OBL measurement was independently evaluated by three senior surgeons (FG, Xu H, and LL) to ensure consensus and accuracy, preventing any bias from the software, and the mean value of their measurements was defined as the ground truth.

### 2.5 Data splitting

Our study developed two OBL prediction algorithms: OBLPA-Net for predicting OBL morphology and a DL-based regression model for estimating OBL area, both utilizing the same dataset. Training and validation data were sourced from the Second Affiliated Hospital of Nanchang University, with strict patient-level splitting implemented to ensure both eyes of the same patient were assigned to the same dataset. To assess model generalizability, two independent datasets were used: external test set 1 from Nanchang Bright Eye Hospital and external test set 2 from Hangzhou Huaxia Eye Hospital (Fig. 2).

**Fig. 1:**
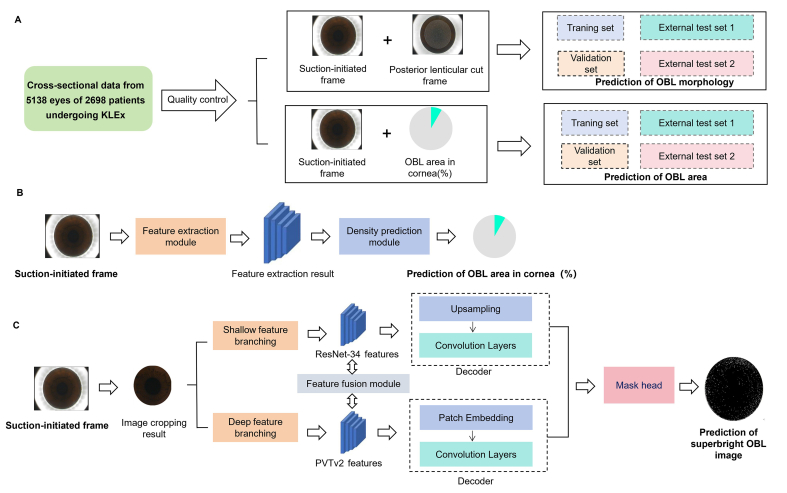
Development and validation of the deep-learning system for predicting OBL morphology and area in KLEx. (A) collecting and splitting data for OBL morphology and area prediction based on frames of KLEx videos. (B) pipeline for OBL morphology prediction. (C) pipeline for OBL area prediction Abbreviations: OBL=opaque bubble layer; KLEx=keratorefractive lenticule extraction.

**Fig. 2:**
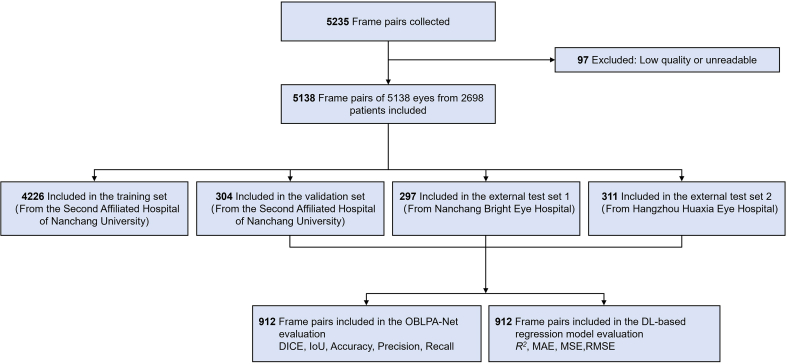
Flow chart of frame quality control and the datasets used in the current study. Abbreviations: IoU=intersection over union; MAE=mean absolute error; MSE=mean squared error; OBL=opaque bubble layer; *R*^2^=*R*-squared; RMSE=root mean squared error.

### 2.6 Image preprocessing

Preprocessing was conducted to isolate the region of interest within the surgical video frames. An elliptical mask was generated to perform semantic segmentation of the posterior lenticular cut frame. The frame was binarized by calculating the mean and SD of intensity within the masked area, and a threshold was applied to delineate high-intensity OBL regions (superbright OBL image). Both the central region of the mask and the corresponding suction-initiated frame were cropped to an oval dimension of 380 × 415 pixels to eliminate background noise and preserve essential visual information.

### 2.7 OBLPA-net architecture for morphology prediction

**Supplementary Fig. S3:**
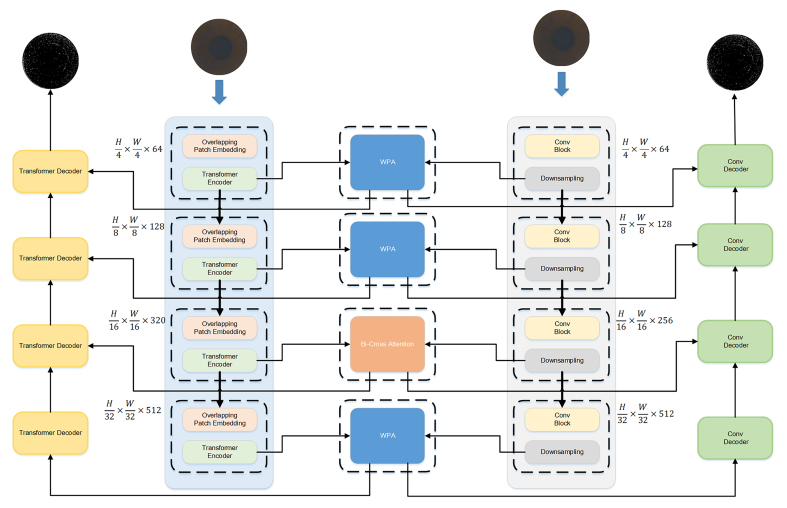
Detailed architecture of the opaque bubble layer perceptual attention network (OBLPA-Net) OBLPA-Net consists of a parallel encoder, parallel decoder, and modules for Bi-Cross and Bi-Win Perception Attention. The parallel encoder has two branches: ResNet-34 pretrained on ImageNet for local information and PVTv2 for global information. Bi-Cross and Bi-Win Perception Attention merge these features during decoding. The final output is an enhanced posterior lenticular cut frame (superbright OBL image).

We developed OBLPA-Net, a specialized DL architecture for OBL morphology prediction. In contrast to conventional segmentation models that typically rely on a single encoder, OBLPA-Net employs a dual-encoder architecture to extract multi-level semantic features (Fig. 1C and Supplementary Figure S3). This architecture comprises two parallel branches: a convolutional neural network (CNN) branch and a Transformer branch. The CNN branch utilizes a ResNet-34 encoder (pretrained on ImageNet) to extract fine-grained local details, such as texture and edge structures.^15^ Concurrently, the Transformer branch employs a PVTv2 backbone (pretrained on ImageNet) to model global contextual information and long-range dependencies.^16^ The PVTv2 utilizes a pyramid structure that progressively reduces feature resolution while increasing semantic abstraction, effectively capturing multi-scale contextual information. Both branches contain four stages to generate feature representations across multiple spatial scales.

To synthesize information from these parallel encoders, we integrated a cross-attention mechanism within the skip connections. This module facilitates the exchange of information between the CNN and Transformer branches, allowing critical cues to be efficiently fused and transmitted to the decoder. This design leverages the complementary strengths of both architectures, yielding richer semantic representations than single-encoder designs. The fused features are subsequently processed by a convolutional decoder for progressive reconstruction. The decoder consists of blocks containing 3 × 3 convolutions, batch normalization, and ReLU activation, with upsampling implemented via transposed convolutions to restore spatial resolution and generate the final pixel-level prediction. To further interpret the spatial focus, we generated heatmaps. Specifically, Gaussian smoothing was applied to the discrete prediction points to obtain a continuous spatial response-intensity distribution. The smoothed result was then normalized, mapped to a heatmap, and overlaid on the suction-initiated frame to visualize the correspondence between high-response regions and anatomical features.

### 2.8 Model predicting the OBL area in cornea

**Supplementary Fig. S4:**
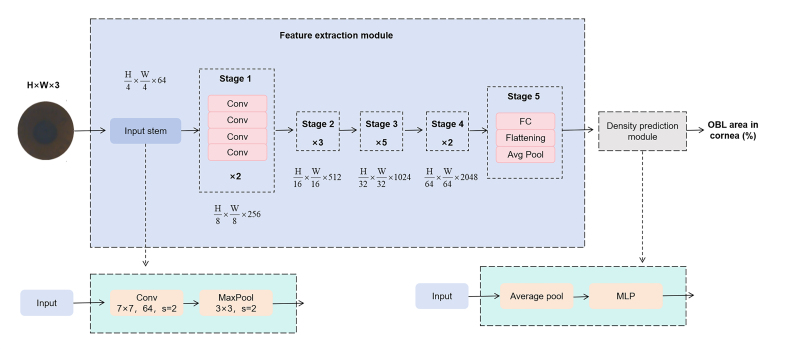
Detailed architecture of the deep learning (DL) based opaque bubble layer (OBL) area prediction regression model The DL-based OBL Prediction Regression Model comprises two main components: the feature extraction module and the density prediction module. The feature extraction module uses the first four blocks of a ResNet-50 backbone pretrained on ImageNet, with parameters frozen during training, to extract a wide range of visual features. The density prediction module includes an average pooling layer and a fully connected layer to predict the final OBL area in the cornea.

The DL-based OBL Prediction Regression Model consisted of two main components: the feature extraction module and the density prediction module (Fig. 1B and Supplementary Figure S4). To extract features, we utilized the first four blocks of the ResNet-50 backbone pre-trained on ImageNet,^15^ with parameters frozen during training. This network effectively extracted a broad range of visual features. The density prediction module included an average pooling layer and a fully connected layer, predicting the final value of the OBL area in the cornea.

### 2.9 Implementation and training strategy

Given the relatively homogeneous illumination and viewpoint of the intraoperative setting, a moderate data augmentation strategy was adopted to enhance model robustness. Random horizontal flipping and random rotation were applied to the training set to simulate minor variations in eye orientation during surgery.

All experiments were implemented using the PyTorch framework on a workstation equipped with an Intel i9-12900K CPU, 62 GB RAM, and an NVIDIA GeForce RTX 3090 GPU (running Ubuntu 22.04). We utilized the AdamW optimizer with both the initial learning rate and weight decay set to 1 × 10^-^^4^. Input images were uniformly resized to 224 × 224 pixels. The models were trained for 200 epochs with a batch size of 8. Task-specific loss functions were adopted: the OBLPA-Net segmentation task was optimized using a composite loss defined as the sum of Dice and IoU loss ($L_{\text{seg}}=L_{\text{Dice}}+L_{\text{IoU}}$), while the regression model was trained using mean squared error loss ($L_{\text{reg}}=\text{MSE}$).

### 2.10 Statistical analysis

The descriptive statistics were presented as the mean (SD) for continuous data and as the number for categorical variables. Independent *t*-tests and Chi-square tests were used to compare the baseline characteristics of participants across all datasets. The inter-observer agreement among the three observers was evaluated using the intraclass correlation coefficient based on a two-way mixed-effects model with absolute agreement. Prediction performance for corneal OBL% was evaluated using *R*-squared (*R*^2^), mean absolute error (MAE), mean squared error (MSE), and root mean squared error (RMSE) with 95% confidence interval (CI). Statistical significance was indicated by a two-tailed *P*-value below 0.05. All analyses were conducted in R (version 4.3.3; https://www.r-project.org/), with code available on GitHub (https://github.com/wuxuanttt/OBLFormer).

## 3 Results

### 3.1 Database description

The two DL models utilized a shared database of 10276 frames from 5138 videos, representing 5138 eyes of 2698 patients. The data were divided into four sets: the training set (4226 eyes from 2317 patients, mean (SD) OBL area of 3.30% (0.62%)), the validation set (304 eyes from 288 patients, mean (SD) OBL area of 3.23% (0.56%)), external test set 1 (297 eyes from 166 patients, mean (SD) OBL area of 3.73% (0.57%)), and external test set 2 (311 eyes from 170 patients, mean (SD) OBL area of 3.78% (0.63%)). Baseline characteristic variations across the sets were also analyzed (Table 1). Supplementary Table S1 showed a good inter-observer agreement in measurements of OBL between the three surgeons.

**Table 1:** Summary of study participants.

	Training set	Validation set	External test set 1	External test set 2	*P*_1_^a^	*P*_2_^b^
KLEx videos	4226	304	297	311	-	
Patients	2317	288	166	170	-	-
Age, years	22.49 (5.56)	22.58 (5.51)	25.28 (5.64)	23.67 (4.95)	<0.001	<0.001
Male/Female	1511/806	196/92	85/86	76/94	<0.001	<0.001
Right/Left	2143/2083	166/138	154/143	159/152	0.704	0.888
OBL in cornea, mean (SD), %	3.30 (0.62)	3.23 (0.56)	3.73 (0.57)	3.78 (0.63)	<0.001	<0.001
Sphere, mean (SD), D	-3.99 (1.62)	-4.00 (1.45)	-3.95 (1.52)	-3.90 (1.46)	0.738	0.355
Cylinder, mean (SD), D	-0.94 (0.53)	-0.96 (0.56)	-0.64 (0.39)	-0.75 (0.42)	<0.001	<0.001
K1, mean (SD), D	42.45 (1.38)	42.43 (1.28)	42.65 (1.36)	42.68 (1.29)	0.007	0.002
K2, mean (SD), D	43.57 (1.46)	43.60 (1.34)	43.73 (1.46)	43.68 (1.36)	0.053	0.147
CCT, mean (SD), μm	546.27 (27.56)	544.05 (24.68)	548.46 (25.86)	551.52 (26.61)	0.269	0.001
Horizontal cornea diameter, mean (SD), mm	11.59 (0.43)	11.58 (0.42)	11.57 (0.39)	11.51 (0.38)	0.401	<0.001
IOP, mean (SD), mmHg	15.76 (2.87)	15.45 (2.67)	14.93 (2.82)	15.24 (2.89)	<0.001	<0.001
Cap thickness (SD), mm	118.96 (3.05)	118.88 (3.16)	119.56 (2.05)	119.74 (1.59)	<0.001	<0.001
Optical Zone, mean (SD), mm	6.49 (0.04)	6.49 (0.05)	6.50 (0.02)	6.50 (0.03)	0.005	0.009
Residual stromal thickness, mean (SD), μm	325.08 (34.41)	322.19 (29.20)	327.46 (31.84)	332.04 (30.03)	0.078	<0.001
Lenticule thickness, mean (SD), μm	102.25 (27.80)	102.96 (26.02)	101.41 (25.77)	99.74 (24.41)	0.615	0.118

**Abbreviations:** CCT=central corneal thickness; D=diopter; IOP=intra-ocular pressure; KLEx=keratorefractive lenticule extraction; OBL=opaque bubble layer; RST=residual stromal thickness; SD=standard deviation.

For continuous variables, the data were expressed as either the mean (SD) and for categorical variables, as percentages.

a Comparison of the demographic variables between training and external test set 1 by independent *t*-tests (Age, OBL in cornea, Sphere, Cylinder, K1, K2, CCT, Horizontal cornea diameter, IOP, Optical Zone, Residual stromal thickness, and Lenticule thickness) or Chi-square tests (Sex and Right/Left eyes).

b Comparison of the demographic variables between training and external test set 2 by independent *t*-tests (Age, OBL in cornea, Sphere, Cylinder, K1, K2, CCT, Horizontal cornea diameter, IOP, Optical Zone, Residual stromal thickness, and Lenticule thickness) or Chi-square tests (Sex and Right/Left eyes).

### 3.2 Performance of the DL-based OBL prediction regression model to predict OBL area in cornea

**Fig. 3:**
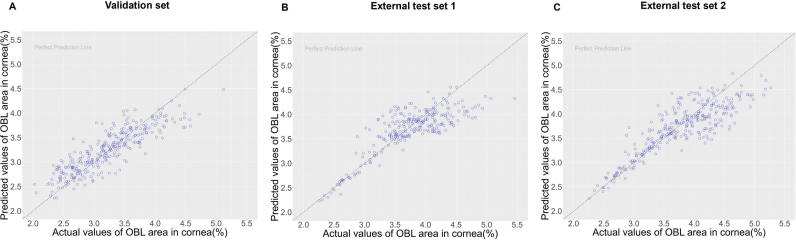
Comparison between the deep learning based regression model predicted and actual opaque bubble layer (OBL) area in cornea (%) in the validation set (A), external test set 1 (B), and external test set 2 (C).

The performance of the regression model in predicting OBL area (%) was shown in Table 2. In the validation set, the model achieved an *R*^2^ of 0.744 (95% CI: 0.687−0.801), an MAE of 0.221 (95% CI: 0.201−0.242), an MSE of 0.082 (95% CI: 0.068−0.095), and an RMSE of 0.286 (95% CI: 0.272−0.299). The generalizability of the model was assessed using external test sets 1 and 2. Scatterplots comparing predicted and actual OBL% values across the validation and test sets are presented in Fig. 3.

**Table 2:** Performance of the OBL prediction models in the validation and external test sets.

	Validation set	External test set 1	External test set 2
DL-based Regression model for predicting OBL% area in cornea			
*R*^2^ (95% CI)	0.744 (0.687−0.801)	0.695 (0.632−0.759)	0.727 (0.669−0.786)
MAE (95% CI)	0.221 (0.201−0.242)	0.229 (0.204−0.254)	0.246 (0.221−0.270)
MSE (95% CI)	0.082 (0.068−0.095)	0.100 (0.081−0.119)	0.108 (0.090−0.126)
RMSE (95% CI)	0.286 (0.272−0.299)	0.317 (0.298−0.335)	0.329 (0.311−0.347)
OBLPA-Net for predicting OBL morphology			
Dice (95% CI)	0.918 (0.915−0.921)	0.924 (0.920−0.927)	0.917 (0.915−0.919)
IoU (95% CI)	0.849 (0.844−0.854)	0.860 (0.854−0.865)	0.850 (0.844−0.855)
Accuracy (95% CI)	0.961 (0.959−0.962)	0.963 (0.961−0.965)	0.960 (0.959−0.962)
Precision (95% CI)	0.907 (0.903−0.910)	0.903 (0.900−0.906)	0.899 (0.897−0.902)
Recall (95% CI)	0.930 (0.927−0.934)	0.946 (0.941−0.951)	0.938 (0.934−0.942)

**Abbreviations:** CI=confidence interval; DL=deep learning; IoU=intersection over union; MAE=Mean Absolute Error; MSE=Mean Squared Error; OBL=opaque bubble layer; *R*^2^=*R*-squared; RMSE=Root Mean Squared Error.

Dice coefficient, IoU, Accuracy, Prediction, and Recall with 95% CI were implemented to assess the performance of OBLPA-Net. Performance in predicting quantitative measurement (OBL%) was performed using *R*^2^, MAE, MSE, and RMSE with 95% CI.

### 3.3 Performances of OBLPA-net to predict OBL morphology

The predictive performance of OBLPA-Net for OBL morphology and distribution was evaluated in Table 2. In the validation set, OBLPA-Net achieved a Dice coefficient of 0.918 (95% CI: 0.915−0.921), IoU of 0.849 (95% CI: 0.844−0.854), accuracy of 0.961 (95% CI: 0.959−0.962), precision of 0.907 (95% CI: 0.903−0.910), and recall of 0.930 (95% CI: 0.927−0.934). The model also demonstrated good generalizability in external test sets (Table 2).

### 3.4 Visualization of the OBL prediction models

**Fig. 4:**
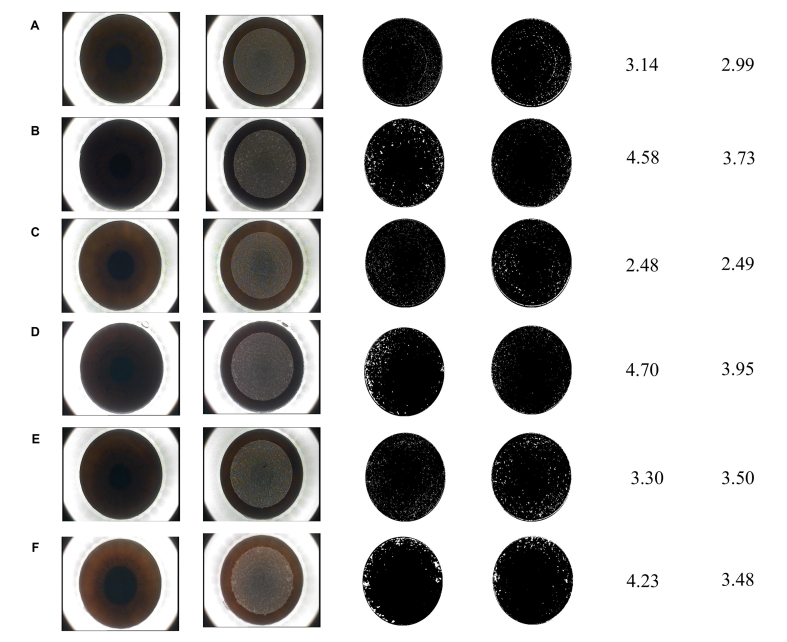
Examples predicted by OBL prediction models in the validation set and external test set 1 and external test set 2. Examples A, C, and E from the validation set, external test set 1, and external test set 2 demonstrated the examples in which both OBL perceptual attention network (OBLPA-Net) and the OBL prediction regression model can accurately predict the superbright OBL image and the OBL area in the cornea (%). Conversely, B, D, and F from the same datasets showed the examples in which the predicted OBL area with significant deviations by the regression model, but can be roughly located in OBLPA-Net. From left to right: suction-initiated frames, posterior lenticular cut frames, real superbright OBL image, predicted superbright OBL image, real OBL%, predicted OBL%. Abbreviations: OBL=opaque bubble layer.

**Supplementary Fig. S5:**
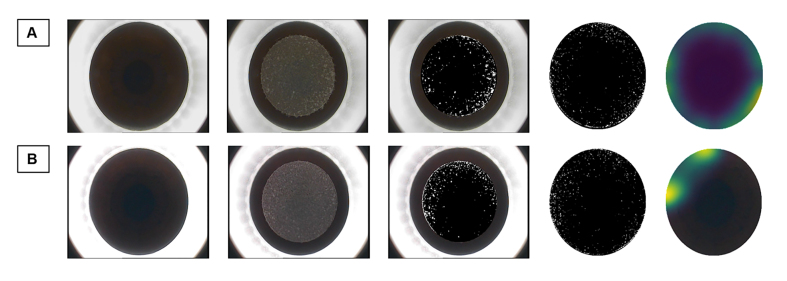
Visualization for OBL morphology prediction Examples A (external test set 1) and B (external test set 2) demonstrate the spatial correspondence between the focus of OBLPA-Net and the actual OBL distribution. Warmer colors in the heatmaps (overlaid on the suction-initiated frames) indicate regions of high model response. From left to right: suction-initiated frame, posterior lenticular cut frame, real superbright OBL image, predicted superbright OBL image, heatmap overlaid on suction-initiated frame.

OBLPA-Net accurately predicted the location and morphology of OBL in both the validation and external test sets, showing high consistency with the actual posterior lenticular cut frame and superbright OBL images (Fig. 4A–C, and E). Even in cases where the predicted OBL% values of the regression model deviated significantly (Fig. 4B–D, and F), OBLPA-Net still identified similar bubble clustering areas. While the majority of the bubble aggregation areas were predicted accurately, some discrepancies remained in regions with sparse bubble distributions between predicted and actual images. Furthermore, heatmaps overlaid on suction-initiated frames revealed that the high-response regions of OBLPA-Net consistently aligned with high-density OBL aggregation areas, particularly in the mid-peripheral cornea (Supplementary Figure S5).

## 4 Discussion

This study introduces two DL models designed to predict the morphology, position, and proportion of OBL in cornea during KLEx surgery. To our knowledge, this is the first machine learning algorithm developed to predict images of intraoperative complications in corneal refractive surgery, with validation conducted across multiple centers, offering potential future support for decision-making in KLEx surgeries.

A distinctive feature of this study is the introduction of suction-initiated frames from KLEx videos to build OBL prediction models, demonstrating superior performance. OBL is a common complication during KLEx and other femtosecond laser surgeries.^17^^,^^18^ Extensive research has indicated corneal parameters (e.g., CCT and corneal curvature), surgical parameters (e.g., cap thickness, lenticule thickness, RST, and laser spot settings), and techniques (e.g., tear-film control and docking technique), are risk factors for OBL during KLEx procedure.^8^, ^9^, ^10^^,^^19^^,^^20^ However, a predictive model for KLEx intraoperative complications based on preoperative parameters has yet to be developed, likely because traditional linear regression fails to capture complex nonlinear interactions.^11^^,^^21^ In addition, preoperative static variables often do not reflect the pathophysiological state during surgery, as the real-time corneal parameters like CCT and biomechanics are influenced by factors such as diurnal variation, hydration, and psychological status.^22^, ^23^, ^24^ For femtosecond laser surgeries, corneal hydration levels affect the quality of lamellar cuts and laser ablation.^25^^,^^26^

While environmental variations (e.g., humidity and temperature) may influence hydration and contribute to OBL distribution shifts across centers, the suction-initiated frame effectively captures this real-time in situ corneal status. This capability ensures model robustness against such inter-center and individual variations, reinforcing the rationale for image-based intraoperative prediction and supporting its potential for broader multi-center application. Furthermore, even though the curved contact glass interface physically masks native corneal curvature and standardized protocols minimize visible decentration, our models maintained robust predictive performance. This suggests that OBLPA-Net may detect high-dimensional visual patterns within the docking interface reflecting subtle corneal tissue responses to suction pressure, which are difficult for human observers to quantify. The specific attention to the mid-peripheral cornea in the suction frame, which spatially corresponds to the subsequent gas migration and high-density OBL accumulation, aligns with this possibility.

For clinical management needs, previous research typically quantified and categorized clinically significant or severe OBL, linking larger areas (e.g., >5%) to complications such as flap tears or increased aberrations.^8^^,^^10^^,^^27^ To address the varying standards in defining it, our system outputs both a quantitative measurement and a morphological prediction on the posterior lenticular cut frame, where OBL is more difficult to dissect. While a universal consensus on an OBL area threshold for surgical intervention is not yet established, the quantitative output provides an objective global index for standardized severity grading and risk assessment. However, morphological prediction, rather than absolute percentage alone, serves as the primary driver of intraoperative decision-making. Concurrently, the morphological prediction offers the spatial information essential for intraoperative decision-making. For example, clusters of OBL can complicate lenticule extractions in clinical practice, particularly in patients with thin lenticules (low myopia) where edge identification is critical, or when located at the 6 and 4 o'clock positions on the posterior side. Highlighting these areas before laser scanning by OBLPA-Net, surgeons, especially in the early stages or those still in training, can consider decisions like re-suction, water absorption, irrigation or even delaying the surgery,^5^^,^^7^^,^^28^ while OBL may depend on factors such as docking technique, tear-film control, or corneal hydration levels.^9^^,^^20^^,^^24^

Our models have some advantages in clinical settings. Firstly, based on the real-time frames of the surgery, our algorithms may form a foundation for future autonomous systems and data-driven improvements in surgical techniques.^29^ This aligns with the broader evolution of refractive surgery, where a recent review highlights the potential of AI-driven intraoperative guidance systems to analyze live surgical feeds and optimize surgical parameters.^30^ Secondly, unlike AI models focused on identifying key anatomical landmarks or effective instrument maneuvers during surgery,^13^^,^^31^ predicting the next step before a key surgical step offers potential flexibility in decision-making,^32^, ^33^, ^34^, ^35^ functioning as a decision-support tool to aid—not replace—surgical judgment regarding future complications. In addition, for operative education, our models provide objective metrics for equitable feedback, and visualizing the predicted OBL morphology may assist trainees in assessing potential surgical risks and guiding procedural decision-making, potentially improving their surgical performance.^36^^,^^37^

This study also has several limitations. Firstly, we prioritized predicting OBL due to its higher frequency compared to events like suction loss.^8^^,^^17^ Future research should extend predictions to other intraoperative complications and long-term outcomes to aid comprehensive decision-making. Secondly, the exclusion of low-quality videos limits applicability to scenarios with poor patient cooperation. Thirdly, due to the rarity of severe OBL in standardized procedures, future model iterations should incorporate data from novice surgeons to improve accuracy for extreme cases. Fourthly, our definition of OBL relied on a standardized image-processing threshold (Mean + 2SD) verified by experts. While this method ensures statistical consistency in identifying high-density bubbles, it may lack biological completeness by underestimating faint or low-density OBLs that do not exceed this threshold. Future studies could address this by employing rigorous pixel-level manual segmentation on raw images. Fifthly, data were derived from fixed laser settings. Since variations in parameters such as higher energy, tighter spot spacing, or thinner corneal caps significantly influence OBL formation,^8^^,^^19^^,^^38^ future validation across diverse configurations is necessary to optimize model generalizability. Sixthly, we did not quantify functional outcomes like dissection time or the usage of additional maneuvers; while our model enhances laser phase predictability, future studies must explicitly link these predictions to operative difficulty. Seventhly, relying solely on suction-initiated frames excludes preoperative clinical parameters (e.g., corneal topography) and other intraoperative visual information (e.g., centering-checked frame). Our future work aims to develop a multimodal fusion network to integrate these heterogeneous data for improved precision. Finally, as our dataset was specific to the Chinese population, future studies should consider diverse demographics and environmental conditions.

## 5 Conclusions

In summary, our study demonstrates the feasibility of using the DL system to predict intraoperative complications before laser scanning in corneal refractive surgery. This provides new possibilities for intraoperative decision-making, improvement of autonomous systems in surgical techniques, and surgical education in future corneal refractive surgery.

## Study approval

The authors confirm that any aspect of the work covered in this manuscript that involved human patients or animals was conducted with the ethical approval of all relevant bodies and the study was performed in accordance with the Declaration of Helsinki, and the protocol was approved by the Ethics Committee of the Second Affiliated Hospital of Nanchang University (approval number: 2023 no. (96)) and registered at ClinicalTrials.gov (NCT06204926).

## Author contributions

The authors confirm contribution to the paper as follows: Yifeng Y: Conceptualization, Formal analysis, Writing - Original draft, Funding acquisition; XW: Conceptualization, Writing - Original draft; FG, Xinghui H, MZ, Xu H, ZZ, TZ, CA, YL, Jingjing X, KY, XX and DS: Data curation; YH and LL: Formal analysis; FX: Validation; Yugen Y: Supervision, Writing - Reviewing and Editing; Jian X: Conceptualization, Supervision, Project administration, Writing - Reviewing and Editing, Funding acquisition. All authors reviewed the results and approved the final version of the manuscript.

## Declaration of generative AI use

The authors declare that no generative AI or AI-assisted technologies were used in the writing process of this manuscript. The deep learning models described in this study form part of the research methodology and are detailed in the Methods section.

## Funding

This study was supported by the 10.13039/501100001809National Natural Science Foundation of China (82260214), the Science and Technology Program of 10.13039/501100020205Jiangxi Provincial Health Commission (202210631, 202110043) and the 10.13039/501100004479Jiangxi Provincial Natural Science Foundation (20212ACB206022).

## Declaration of competing interest

The authors declare that they have no known competing financial interests or personal relationships that could have appeared to influence the work reported in this paper.
