# Supplementary material for: Deep learning models for predicting opaque bubble layer morphology of keratorefractive lenticule extraction before laser scanning
Source: Adv Ophthalmol Pract Res. 2026 Feb 13;6(3):211–8. doi: 10.1016/j.aopr.2026.02.003 (PMC13311923; doi:10.1016/j.aopr.2026.02.003)
Supplement: Multimedia component 1 [file mmc1.pdf]

Supplementary Table S1. Inter-observer agreement for OBL area measurement across datasets

Dataset	No. of frames (n)	OBL area in cornea (%)			ICC	95% CI
		F.G.	Xu.H.	L.L.		
Training set	4226	3.32 ± 0.63	3.28 ± 0.63	3.31 ± 0.64	0.929	0.925 - 0.933
Validation set	304	3.19 ± 0.59	3.26 ± 0.63	3.25 ± 0.50	0.936	0.921 - 0.949
External test set 1	297	3.69 ± 0.60	3.70 ± 0.56	3.80 ± 0.53	0.947	0.913 - 0.966
External test set 2	311	3.72 ± 0.55	3.82 ± 0.72	3.79 ± 0.66	0.926	0.906 - 0.942

Abbreviations: CI= Confidence interval; ICC= intraclass correlation coefficient; OBL = opaque bubble layer

OBL area in cornea were expressed as the mean (SD). ICC estimates and 95% CIs were calculated using a two-way mixed-effects model based on absolute agreement. Evaluators F.G., Xu.H., and L.L. correspond to the three senior surgeons described in the Methods.
